# Distribution and Coexistence of Myoclonus and Dystonia as Clinical Predictors of *SGCE* Mutation Status: A Pilot Study

**DOI:** 10.3389/fneur.2016.00072

**Published:** 2016-05-13

**Authors:** Rodi Zutt, Joke M. Dijk, Kathryn J. Peall, Hans Speelman, Yasmine E. M. Dreissen, Maria Fiorella Contarino, Marina A. J. Tijssen

**Affiliations:** ^1^Department of Neurology, University Medical Center Groningen, Groningen, Netherlands; ^2^Department of Neurology, Academic Medical Center, University of Amsterdam, Amsterdam, Netherlands; ^3^MRC Centre for Neuropsychiatric Genetics and Genomics, Institute of Psychological Medicine and Clinical Neurosciences, School of Medicine, Cardiff University, Cardiff, UK; ^4^Department of Neurology, Haga Teaching Hospital, Den Haag, Netherlands

**Keywords:** myoclonus–dystonia, myoclonus, dystonia, *SGCE* mutations, motor characteristics

## Abstract

**Introduction:**

Myoclonus–dystonia (M–D) is a young onset movement disorder typically involving myoclonus and dystonia of the upper body. A proportion of the cases are caused by mutations to the autosomal dominantly inherited, maternally imprinted, epsilon-sarcoglycan gene (*SGCE*). Despite several sets of diagnostic criteria, identification of patients most likely to have an *SGCE* mutation remains difficult.

**Methods:**

Forty consecutive patients meeting pre-existing diagnostic clinical criteria for M–D underwent a standardized clinical examination (20 *SGCE* mutation positive and 20 negative). Each video was reviewed and systematically scored by two assessors blinded to mutation status. In addition, the presence and coexistence of myoclonus and dystonia was recorded in four body regions (neck, arms, legs, and trunk) at rest and with action.

**Results:**

Thirty-nine patients were included in the study (one case was excluded owing to insufficient video footage). Based on previously proposed diagnostic criteria, patients were subdivided into 24 “definite,” 5 “probable,” and 10 “possible” M–D. Motor symptom severity was higher in the SGCE mutation-negative group. Myoclonus and dystonia were most commonly observed in the neck and upper limbs of both groups. Truncal dystonia with action was significantly seen more in the mutation-negative group (*p* < 0.05). Coexistence of myoclonus and dystonia in the same body part with action was more commonly seen in the mutation-negative cohort (*p* < 0.05).

**Conclusion:**

Truncal action dystonia and coexistence of myoclonus and dystonia in the same body part with action might suggest the presence of an alternative mutation in patients with M–D.

## Introduction

Myoclonus–dystonia (M–D) is a rare movement disorder, characteristically with onset in the first two decades of life (1). Motor features are typically of myoclonic jerks, predominantly involving the upper body, although also involving the lower limbs, face, and larynx in up to a quarter of cases (2–4). The dystonic component most frequently involves the neck and upper limbs (writer’s cramp) (5, 6). Both the myoclonus and dystonia may be exacerbated by posture, action, or stress. Presentation and progression of motor symptoms may vary, ranging from an early childhood-onset form starting with upper body or lower limb involvement and progressing to upper limbs involvement to a later-onset form, with predominant upper body symptoms and frequent cervical involvement. The clinical course can be stable or show a progressive course, with increasing severity and/or spreading of symptoms (7). Alcohol consumption is widely reported to improve motor symptoms, particularly the myoclonus, resulting in excess alcohol consumption in some cases (8, 9). Several studies have also identified psychiatric symptoms in M–D cohorts, including anxiety, panic attacks, and obsessive–compulsive disorder (10–12).

Myoclonus–dystonia is inherited in an autosomal dominant fashion with mutations in the maternally imprinted *SGCE* gene (DYT11) observed in a proportion of cases (13–15). At present, clinical discrimination of *SGCE* mutation-positive from mutation-negative M–D cases remains difficult. Previous studies have shown the frequency of *SGCE* mutations in M–D cohorts to vary between 21 and 85% dependent on the inclusion criteria employed (3, 4, 6, 16–19). Several factors have been proposed as predictors of an *SGCE* mutation, including motor symptom onset <20 years, a positive family history of a similar movement disorder, and comorbid psychiatric symptoms (19–21).

A classification system has been developed to determine the likelihood of an *SGCE* mutation in individual cases, with subgroups “definite,” “probable,” or “possible,” based on the distribution of motor symptoms, age at onset, and presence or absence of a family history (Table S1 in Supplementary Material) (18). Refinement of these diagnostic criteria has been proposed to include a positive family history with specific paternal transmission and normal brain imaging (5), or the combination of young onset motor symptoms with psychiatric features (20). However, it remains difficult to identify those patients most likely to have an *SGCE* mutation.

The aim of this study is to determine whether the characteristics of motor signs observed during clinical examination can be of help in identifying carriers of a *SGCE* mutation. Particular emphasis was placed on whether coexistence of myoclonus and dystonia in the same body part was helpful in distinguishing those with and without an *SGCE* mutation. We hypothesized that *SGCE* mutation-negative patients with “jerky dystonia” would more often present with jerky movements superimposed on the dystonic posture in the same body part, whereas for those with an *SGCE* mutation the myoclonus and dystonia would be evident independently and in different body regions.

## Materials and Methods

Following informed consent, 40 consecutive M–D patients from the movement disorders service at the Academic Medical Center, Amsterdam, The Netherlands were recruited for the study (20 *SGCE* positive and 20 *SGCE* negative). Participants were divided into one of the three diagnostic categories, “definite,” “probable,” and “possible,” according to previously proposed diagnostic criteria (Table S1 in Supplementary Material). All participants underwent a videotaped clinical examination, which in the majority of cases followed a standardized protocol (*n* = 31), and the remaining cases were examined as part of routine clinical practice (*n* = 9). Each videotaped examination was subsequently assessed by two of the three independent movement disorder experts (Maria Fiorella Contarino, Hans Speelman, and Joke M. Dijk), blinded to the genetic status of the participant.

The motor section of the Burke–Fahn–Marsden Dystonia Rating Scale (BFMDRS) and sections 2 (myoclonus at rest) and 4 (action myoclonus) of the Unified Myoclonus Rating Scale (UMRS) were used to assess motor symptom severity (22). In addition, the presence and coexistence of myoclonus and dystonia were recorded in four body regions (neck, arms, legs, and trunk), at rest and with action. In the absence of adequate video footage to allow evaluation of a specific body region in individual patients, the score for this region was omitted.

The Ethical Board of the Academic Medical Center of Amsterdam approved the study.

### Statistical Analysis

Clinical features were analyzed using the Fisher’s exact test or Student’s *t*-test where appropriate. Inter-rater reliability was assessed using intraclass correlation coefficients (ICCs) (two way mixed, consistency, and average measures). ICC results were further classified as; 0.91–1: excellent, 0.71 and 0.9: good, 0.51 and 0.70: moderate, <0.5: poor, and <0.3: very poor (23). The inter-rater reliability of the new evaluation tool was reported both in absolute agreement and percentage of agreement between raters.

## Results

### Demographic Characteristics

A full summary of the demographic characteristics of this cohort is reported in Table 1. Due to the consecutive nature of recruitment, mutation-positive and -negative groups were not matched for gender and age at the onset of motor symptoms. The *SGCE* mutation-positive cohort included a greater number of cases with motor symptom onset <20 years and a positive family history. There were no significant differences in demographic characteristics between the groups.

**Table 1:** **Demographic characteristics**.

	*SGCE* mutation positive Proband only cohort (*n* **=** 13)	*SGCE* mutation positive All patients (*n* **=** 19)	*SGCE* mutation negative cohort (*n* **=** 20)	*SGCE* positive vs. *SGCE* negative (*p*-value)
Gender				
Male/female	5/8	9/10	5/15	0.19
Age median (range)	40 (15–60)	41 (15–75)	36 (14–61)	0.56
Age at onset				
≤20 years/>20 years	12/1	17/2	12/8	0.07
Symptom at onset				
Myoclonus	9	13	15	0.73
Dystonia	1	3	5	0.70
Myoclonus and dystonia	3	3	0	0.11
Alcohol responsiveness				
Responsive	5	5	6	0.23
Unresponsive	0	0	4	
Unknown	8	14	10	
Family history				
Positive/negative	13/0	…	8/12	0.00
“Grunewald classification”				
“Definite”	12	17	7	0.00
“Probable”	0	1	4	0.34
“Possible”	1	1	9	0.01

Overall, 39 patients (25F: 14M) with a clinical M–D phenotype were included in the study. Nineteen had an *SGCE* mutation (one mutation-positive case was excluded owing to insufficient video footage) and 20 patients were mutation negative. Median age at examination was 39 years (range: 14–75). Myoclonus was the presenting feature in 28 cases, dystonia in 8 and both myoclonus and dystonia were observed at symptom onset in 3. Details of the cognitive and psychological characteristics of this cohort have been published elsewhere (12).

The *SGCE* mutation-positive cohort (*n* = 19, 10F: 9M) included 13 probands and had a median age at examination of 41 years (range: 15–75). Seventeen cases had onset of symptoms <20 years of age with single cases developing motor symptoms in each of the 30- to 40-year and 40- to 50-year age brackets. Applying Grunewald diagnostic criteria, this group was further subdivided into 17 “definite,” 1 “probable,” and 1 “possible” cases.

The *SGCE* mutation-negative cohort (*n* = 20, 15F: 5M) had a median age at examination of 36 years (range: 14–61). Motor symptom onset was <20 years in 12 cases, 30–40 years in 4 cases, 40–50 years in 3 cases, and >50 years in a single participant. With application of the same diagnostic criteria, this group was sub-divided into 7 “definite,” 4 “probable,” and 9 “possible.”

Due to a sub-optimal video footage, 11/76 (at rest) and 11/76 (with action) dystonia and 2/76 (at rest) and 3/76 (with action) myoclonus video assessment sections were scored as missing in the *SGCE* mutation-positive cohort. In the mutation-negative group, 2/80 (at rest) and 3/80 (with action) dystonia while none of the myoclonus assessment sections incomplete.

### Distribution of Symptoms

Myoclonus and dystonia were most commonly observed in the neck and arms in both mutation positive and negative groups. Comparison of the *SGCE* mutation-positive probands and the mutation-negative group identified significant difference with truncal dystonia during action [8/19 (*SGCE* mutation-negative) 0/13 (*SGCE* mutation-positive probands only); *p* = 0.01, OR 0.01, 95% CI (0.00, 0.74)]. This difference was preserved when extended to include the entire mutation-positive group: truncal dystonia [8/19 (*SGCE* mutation-negative) 1/17 (*SGCE* mutation-positive); *p* = 0.02, OR 0.09, 95% CI (0.00, 0.88)] (Tables 2 and 3; Figure S1 in Supplementary Material).

**Table 2:** **Distribution of myoclonus at rest and with action**.

Myoclonus	*SGCE* positive All (*n* **=** 19)	*SGCE* positive Proband only (*n* **=** 13)	*SGCE* negative (*n* **=** 20)	Statistical comparison All/negative, *p*-value (OR; 95% CI)	Statistical comparison Proband only/negative, *p*-value (OR; 95% CI)
Rest
Neck	12	9	13	1.00 (0.92; 0.21, 4.15)	1.00 (1.21; 0.22, 6.97)
Upper limbs	5	4	13	0.05 (0.22; 0.04, 1.09)	0.08 (0.24; 0.04, 1.32)
Trunk	7	5	3	0.07 (4.41; 0.74, 29.07)	0.12 (4.05; 0.59, 30.64)
Lower limbs	3	2	6	1.00 (0.70; 0.10, 4.41)	0.68 (0.52; 0.06, 4.00)
Action					
Neck	10	8	16	0.29 (0.42; 0.07, 2.30)	0.43 (0.50; 0.07, 3.30)
Upper limbs	12	9	14	1.00 (0.86; 0.18, 4.14)	1.00 (0.96; 0.17, 5.68)
Trunk	6	4	6	1.00 (1.27; 0.26, 6.29)	1.00 (1.04; 0.18, 6.02)
Lower limbs	3	2	3	0.67 (1.55; 0.20, 12.33)	1.00 (1.13; 0.11, 10.83)

*Fisher’s exact test was used for statistical comparison*.

**Table 3:** **Distribution of dystonia at rest and with action**.

Dystonia	*SGCE* positive All (*n* **=** 19)	*SGCE* positive Proband only (*n* **=** 13)	*SGCE* negative (*n* **=** 20)	Statistical comparison All/negative *p*-value (OR; 95% CI)	Statistical comparison Proband only/negative *p*-value (OR; 95% CI)
Rest					
Neck	14	11	19	0.17 (0.18; 0.01, 2.14)	0.55 (0.29; 0.01, 4.90)
Upper limbs	2	2	2	1.00 (1.20; 0.10, 14.07)	1.00 (1.64; 0.14, 19.91)
Trunk	0	0	4	NA	NA
Lower limbs	1	1	2	1.00 (0.71; 0.02, 12.05)	1.00 (0.85; 0.03, 14.84)
Action					
Neck	7	5	16	0.04 (0.19; 0.03, 1.04)	0.05 (0.18; 0.03, 1.10)
Upper limbs	6	5	11	0.21 (0.41; 0.09, 1.84)	0.48 (0.51; 0.10, 2.63)
Trunk	1	0	8	**0.02 (0.09; 0.00, 0.88)**	**0.01 (0.00; 0.00, 0.74)**
Lower limbs	3	3	6	1.00 (0.73; 0.11, 4.43)	0.69 (0.55; 0.08, 3.44)

*Key: statistically significant differences (*p* < 0.05) between *SGCE* mutation-positive and -negative groups are highlighted in bold*.

*NA, not applicable*.

*Fisher’s exact test was used for statistical comparison*.

### Coexistence of Myoclonus and Dystonia

At rest, there was no significant difference between the proband-only mutation-positive group and those without an *SGCE* mutation in the coexistence of myoclonus and dystonia in the same body part, either overall or by individual body part. Overall assessment with action showed a significant difference between the two groups [*p* = 0.01, OR = 0.11, 95% CI (0.01, 0.73)], being the coexistence more common in the mutation-negative group, although no difference was observed between individual body parts. Inclusion of the entire mutation-positive cohort showed similar results overall [*p* = 0.01, OR 0.13, 95% CI (0.02, 0.71)] and a trend toward significance when examining the cervical region [*p* = 0.09, OR = 0.26, 95% CI (0.05, 1.26)]. A full summary of the rates and distribution of coexistent myoclonus and dystonia can be seen in Table 4.

**Table 4:** **Comparison of co-existent myoclonus and dystonia in the same body region in *SGCE* mutation-positive and -negative cohorts**.

Myoclonus and dystonia	*SGCE* positive All (*n* **=** 19)	*SGCE* positive Proband only (*n* **=** 13)	*SGCE* negative (*n* **=** 20)	Statistical comparison All/negative *p*-value (OR; 95% CI)	Statistical comparison Proband only/negative *p*-value (OR; 95% CI)
Rest					
Overall	10	8	15	0.19 (0.37; 0.08, 1.73)	0.46 (0.53; 0.09, 3.05)
Neck	10	8	13	0.74 (0.67; 0.15, 3.01)	1.00 (0.86; 0.16, 4.63)
Upper limbs	0	0	2	NA	NA
Trunk	0	0	0	NA	NA
Lower limbs	1	1	1	1.00 (1.50; 0.04, 62.14)	1.00 (1.80; 0.04, 75.80)
Action					
Overall	8	5	17	**0.01 (0.13; 0.02, 0.71)**	**0.01 (0.11; 0.01, 0.73)**
Neck	6	5	14	0.09 (0.26; 0.05, 1.26)	0.15 (0.31; 0.05, 1.71)
Upper limbs	5	4	8	0.51 (0.58; 0.12, 2.74)	0.72 (0.67; 0.12, 3.65)
Trunk	1	0	4	0.34 (0.23; 0.01, 2.75)	0.13 (0.00; 0.00, 2.21)
Lower limbs	0	0	0	NA	NA

*Key: statistically significant differences (*p* < 0.05) between *SGCE* mutation-positive and -negative groups are highlighted in bold*.

*NA, not applicable*.

*Fisher’s exact test was used for statistical comparison*.

### Severity of Myoclonus and Dystonia with Use of BFMDRS and UMRS Rating Scales

The median total BFMDRS score was significantly higher in the *SGCE* mutation negative group [6/120 (range: 4–47)] vs. 3.5/120 (range: 0–11) than in the mutation-positive group (*p* < 0.05). A higher median UMRS total score was also observed in the *SGCE*-negative patients [25/240 (range: 0–92)] compared to 14.5/240 (range: 0–80) in the mutation-positive group (*p* > 0.05), although this difference was not statistically significant.

### Inter-Rater Agreement

Two assessors evaluated the *SGCE* mutation-negative group using both BFMDRS and UMRS rating scales, achieving an inter-rater concordance of “good” [ICC BFMDRS = 0.91 (95% CI: 0.74–0.97)/ICC UMRS = 0.87 (95% CI: 0.60–0.96)]. Each rating scale was scored by a single assessor during evaluation of the mutation-positive patients (Table S2 in Supplementary Material). In evaluating co-occurrence of myoclonus and dystonia, overall agreement between the two assessors was 88% at rest and 84% with action. Evaluation of individual body parts showed the strongest concordance when assessing the truncal region (94%, 34/36) and the lowest rate of agreement when evaluating movements of the neck with action (64%, 23/36). A summary of the positive agreement between assessors can be seen in Table S3 in Supplementary Material.

## Discussion

This study examined the distribution and coexistence of myoclonus and dystonia, at rest and with action, as a predictive factor in determining the presence of an *SGCE* mutation in patients with an M–D phenotype. We have demonstrated that truncal dystonia and coexistence of myoclonus and dystonia in the same body region with action are more frequently observed in those without an *SGCE* mutation.

Application of the Grunewald diagnostic criteria to this study cohort did not clearly distinguish between those with and without an *SGCE* mutation. Seven on those without a mutation were deemed to be in the “definite” diagnostic category, while a two individuals with mutations were placed, one each, in the “probable” and “possible” groups. In keeping with the current diagnostic criteria, myoclonus and dystonia were most frequently observed in the neck and arms of both mutation positive and negative cohorts, with onset of symptoms <20 years being more frequent in those with an *SGCE* mutation (17/19 vs. 12/20) (5, 18). A positive family history was more frequently observed among those with an *SGCE* mutation and therefore increased the number of cases in the “definite” diagnostic category. The mutation-positive cohort consisted of 13 probands with an additional 6 affected cases recruited from two families, reflecting an inherent recruitment bias from a specialist tertiary movement disorder service. It could also be argued that by recruiting multiple members of the same kindred, additional genetic and environmental factors may also be contributing to their motor phenotype. However, little difference in results was observed when comparing both the proband only and complete *SGCE* mutation-positive cohort to the mutation-negative group, suggesting that any potential additional factors had little effect in the outcome of this study. In addition, multiple case reports and case series have demonstrated significant intra-familial motor variability among those with *SGCE* mutations (4).

It is worth mentioning that mutations in other genes, including KCTD17, THD, and RELN, have been recently associated with M–D, although confirmation in a larger number of families is still needed (24–26). Available data suggest that the phenotype associated with these mutations might slightly differ from that associated with SGCE mutation. For example the *KCTD17* gene mutation is characterized by a milder myoclonus affecting the upper limbs and progressive dystonia spreading from the craniocervical region to other sites. The patients in our cohort were not screened for these mutations, which could potentially account for some of the SCGE-negative cases.

Although multiple previous reports have commented on worsening of motor features with action in M–D cohorts, none of the previous studies have directly compared the nature and frequency of the movement disorder between an *SGCE* mutation-positive cohort and a suitable mutation-negative control group, both at rest and with action. Overall, no difference between the two groups was observed at rest; however, coexistence of myoclonus and dystonia in the same body area was significantly more frequent with action in the mutation-negative cohort (*p* < 0.05) with a trend toward significance observed in the cervical region with action (*p* = 0.09). These observations of coexistent myoclonus and dystonia in the same body region in those without an *SGCE* mutation may reflect a “jerky” dystonia rather than myoclonus. These two forms of hyperkinesias are known to be difficult to differentiate, both in clinical practice and assessment of videotaped examination. It may be contributory to include neurophysiological testing in future studies to aid in differentiating between these two forms of movement disorder (27).

Multi-rater comparison of clinical cases can potentially result in significant variability of clinical opinion. Overall inter-rater agreement between the movement disorder specialists involved in this study was good. Disparities in scoring were predominantly observed in the cervical region where dystonic “overflow” or movement of other body parts can cause diagnostic difficulty. These results highlight the notoriously difficult task of hyperkinetic movement disorder phenomenology, particularly when two or more movement disorder subtypes may coexist in the same body region. This study can be regarded as a pilot study due to the relatively small number of patients in each study group ultimately preventing further and more elaborate statistic analysis of the available results. Future studies will require large, multicenter collaboration in order to enable recruitment of sufficiently large and diverse cohorts to allow definitive conclusions to be drawn.

## Conclusion

The results of this pilot study suggest that the presence of truncal dystonia and the coexistence of myoclonus and dystonia in the same body region with action reduce the likelihood of *SGCE* mutation in patients with an M–D phenotype. Larger series are needed to confirm our preliminary findings before they can be translated into clinical practice. These results highlight the importance of examining movement disorders both at rest and with action during clinical assessment, particularly, when selecting those patients to undergo specific genetic testing.

## Author Contributions

All authors listed have made substantial, direct, and intellectual contribution to the work and approved it for publication.

## Conflict of Interest Statement

The authors declare that the research was conducted in the absence of any commercial or financial relationships that could be construed as a potential conflict of interest.
