# Supplementary material for: Distribution and Coexistence of Myoclonus and Dystonia as Clinical Predictors of SGCE Mutation Status: A Pilot Study
Source: Front Neurol. 2016 May 13;7:72. doi: 10.3389/fneur.2016.00072 (PMC4865489; doi:10.3389/fneur.2016.00072)
Supplement: Supplementary file 1 [file table_1.docx]

**Supplementary Table 1: Grunewald criteria**

| **Grunewald criteria [15]** | | |  |  |  |  |  |
| --- | --- | --- | --- | --- | --- | --- | --- |
| Definite' |  | Early onset myoclonus and dystonia | | | |  |  |
| M-D | OR | Isolated myoclonus predominantly in the upper body half | | | | | |
|  | AND | Positive family history for myoclonus and/or dystonia | | | | | |
|  |  |  |  |  |  |  |  |
| Probable' |  | Early onset myoclonus and dystonia | | | |  |  |
| M-D | OR | Isolated myoclonus predominantly in the upper body half | | | | | |
|  |  |  |  |  |  |  |  |
| Possible' |  | ‘Jerky dystonia’ of neck | | |  |  |  |
| M-D | OR | Isolated jerky movements of variable distribution | | | | |  |
|  | OR | Signs of dystonia and/or myoclonus in lower body half | | | | | |
|  | OR | No response to alcohol | | |  |  |  |
