# Supplementary material for: Distribution and Coexistence of Myoclonus and Dystonia as Clinical Predictors of SGCE Mutation Status: A Pilot Study
Source: Front Neurol. 2016 May 13;7:72. doi: 10.3389/fneur.2016.00072 (PMC4865489; doi:10.3389/fneur.2016.00072)
Supplement: Supplementary file 2 [file table_2.docx]

**Supplementary Table 2: Comparison of assessor BFMDRS and UMRS scores in SGCE**

**mutation positive and negative cohorts**

|  | |  | Median | Range | SD | ICC |
| --- | --- | --- | --- | --- | --- | --- |
| BFMDRS | |  |  |  |  | (95% CI) |
| Assessor 1 | | |  |  |  |  |
| Overall |  | | 6,00 | 0 - 47 | 9,54 |  |
| *SGCE*-positive | | | 3,50 | 0 - 11 | 3,25 |  |
| *SGCE*-negative | | | 6,00 | 4 - 47 | 12,16 |  |
| Assessor 2 | | |  |  |  |  |
| *SGCE*-negative | | | 7,50 | 2 - 32 | 7,20 | 0,91 (0,74 - 0.97) |
| UMRS |  | |  |  |  |  |
| Assessor 1 | | |  |  |  |  |
| Overall |  | | 21,00 | 0 - 92 | 23,22 |  |
| *SGCE*-positive | | | 14,50 | 0 - 80 | 18,31 |  |
| *SGCE*-negative | | | 25,00 | 0 - 92 | 25,16 |  |
| Assessor 2 | | |  |  |  |  |
| *SGCE*-negative | | | 16,50 | 0 - 73 | 20,16 | 0,87 (0,60 - 0,96) |
