# Supplementary material for: Distribution and Coexistence of Myoclonus and Dystonia as Clinical Predictors of SGCE Mutation Status: A Pilot Study
Source: Front Neurol. 2016 May 13;7:72. doi: 10.3389/fneur.2016.00072 (PMC4865489; doi:10.3389/fneur.2016.00072)
Supplement: Supplementary file 3 [file table_3.docx]

**Supplementary Table 3: Inter-rater agreement of the co-existence of myoclonus and dystonia**

| **Rater 1**  **vs rater 2** | **Neck** | | **Arms** | | **Trunk** | | **Legs** | | **Total** | |
| --- | --- | --- | --- | --- | --- | --- | --- | --- | --- | --- |
|  | **absolute agreement** | **percentage**  **(%)** | **absolute agreement** | **percentage**  **(%)** | **absolute agreement** | **percentage**  **(%)** | **absolute agreement** | **percentage**  **(%)** | **absolute agreement** | **percentage**  **(%)** |
| **Rest** | 31/38 | 82% | 32/37 | 86% | 33/35 | 94% | 29/32 | 91% | 125/142 | 88% |
| **Action** | 23/36 | 64% | 32/38 | 84% | 34/36 | 94% | 30/32 | 94% | 119/142 | 84% |
