# Supplementary figures and images for: Distribution and Coexistence of Myoclonus and Dystonia as Clinical Predictors of SGCE Mutation Status: A Pilot Study
Source: Front Neurol. 2016 May 13;7:72. doi: 10.3389/fneur.2016.00072 (PMC4865489; doi:10.3389/fneur.2016.00072)

Figure 1: Body distribution of myoclonus and dystonia

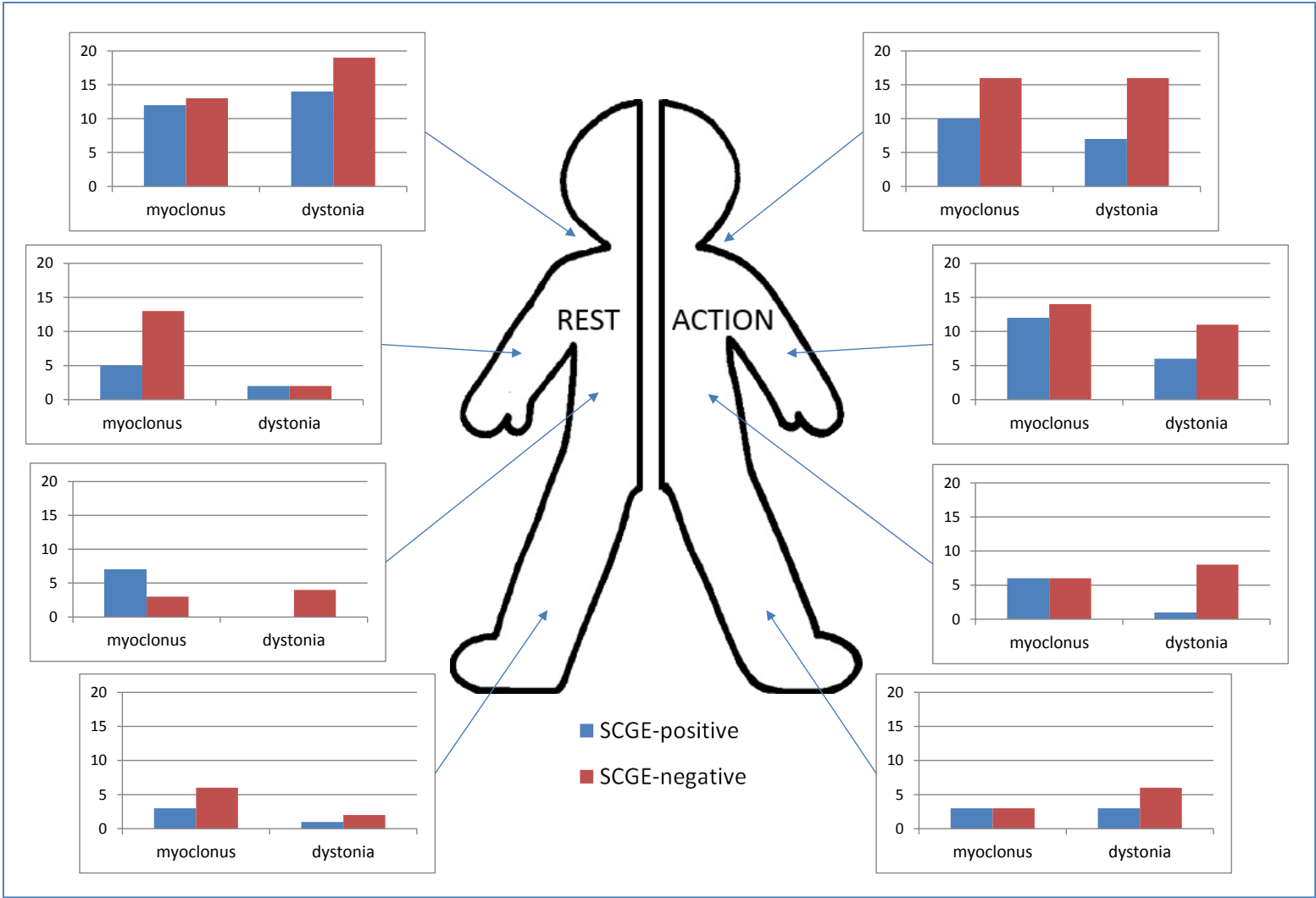

Supplement: Supplementary file 4 [file image_1.pdf]
